# *In vitro* performance of cost differentiated ceftriaxone brands against *Escherichia coli*: insights from a tertiary referral hospital in Mbeya, Tanzania

**DOI:** 10.1093/jacamr/dlae162

**Published:** 2024-10-14

**Authors:** Anthony Nsojo, Christopher Mbotwa, Linus Rweyemamu, Godlove Mbwanji, Frank Wilson, Lutengano George, Davance Mwasomola, Clement N Mweya, Issakwisa Mwakyula

**Affiliations:** Research Unit, Mbeya Zonal Referral Hospital, P. O. Box 419, Mbeya, Tanzania; Mbeya College of Health and Allied Sciences, University of Dar es Salaam, P. O. Box 608, Mbeya, Tanzania; Mbeya College of Health and Allied Sciences, University of Dar es Salaam, P. O. Box 608, Mbeya, Tanzania; Mbeya College of Health and Allied Sciences, University of Dar es Salaam, P. O. Box 608, Mbeya, Tanzania; Research Unit, Mbeya Zonal Referral Hospital, P. O. Box 419, Mbeya, Tanzania; Mbeya College of Health and Allied Sciences, University of Dar es Salaam, P. O. Box 608, Mbeya, Tanzania; Research Unit, Mbeya Zonal Referral Hospital, P. O. Box 419, Mbeya, Tanzania; Mbeya College of Health and Allied Sciences, University of Dar es Salaam, P. O. Box 608, Mbeya, Tanzania; Research Unit, Mbeya Zonal Referral Hospital, P. O. Box 419, Mbeya, Tanzania; Research Unit, Mbeya Zonal Referral Hospital, P. O. Box 419, Mbeya, Tanzania; Mbeya College of Health and Allied Sciences, University of Dar es Salaam, P. O. Box 608, Mbeya, Tanzania; Mbeya Medical Research Centre, National Institute for Medical Research, P. O. Box 2410, Mbeya, Tanzania; Research Unit, Mbeya Zonal Referral Hospital, P. O. Box 419, Mbeya, Tanzania; Mbeya College of Health and Allied Sciences, University of Dar es Salaam, P. O. Box 608, Mbeya, Tanzania

## Abstract

**Background:**

In Tanzania, ceftriaxone is one of the most commonly prescribed antibiotics. However, there is quite a significant variation in cost for numerous ceftriaxone brands, leading to the perception that pricier options are more effective. Yet, limited empirical data support this perception.

**Methods:**

Five ceftriaxone brands with a wide price range were tested *in vitro* against a ceftriaxone-sensitive *Escherichia coli* clinical isolate using microdilution and spectrophotometry. Brands were evaluated across a spectrum of concentrations. Bacterial growth inhibition was measured using optical density. Analysis of variance was used to compare the bacterial optical densities among the brands.

**Results:**

All brands were comparable at all tested concentrations, with peak inhibition above 1.95 mg/L.

**Conclusions:**

Despite significant price disparities, low-cost and high-cost ceftriaxone brands demonstrated similar *in vitro* performance against *E. coli*. This challenges the notion that higher-priced options offer better performance. Further, *in vivo* studies are recommended to validate these findings.

## Introduction

Antimicrobials are pivotal in the fight against bacterial infections, contributing significantly to global public health outcomes.^1^ However, antimicrobial resistance (AMR) poses a formidable threat to global public health, necessitating optimal use of available antibiotics. With the proliferation of generic formulations, multiple brands of antimicrobials are now available in many markets at varying costs. This is especially true in developing countries like Tanzania, where generic drugs co-exist with low-cost alternative brands. However, perceptions that higher-priced options are superior in performance prevail in these markets, influencing prescribing behaviours.^2–4^ While it is known that the direct and indirect effects of low-quality drugs have significant health and economic impact, no part of the world escapes the challenge^5^; the use of sub-standard antimicrobials has negative consequences for users and is one of the drivers of AMR.^5^ There is an urgent need to investigate if the evidence supports this price–performance association.^6,7^

Ceftriaxone, a third-generation cephalosporin, is pivotal in treating invasive infections caused by Gram-negative bacteria like uropathogenic *Escherichia coli*. With its broad-spectrum coverage, bactericidal action, and favourable safety profile, ceftriaxone is among the most commonly used empiric and definitive therapies for bacterial infections in Tanzania.^8,9^ In the Tanzanian pharmaceutical market, ceftriaxone is available under various brands at prices ranging widely from less than 1 USD to over 20 USD per gramme.^10^ Despite such price variations, data directly comparing their *in vitro* antibacterial performance of low-cost versus high-cost ceftriaxone formulations are lacking.^11,12^

Perceptions that low-cost antimicrobials are inferior could prevent the uptake of generics, straining healthcare budgets and threatening access.^4,13^ Scientifically, assessing if the evidence supports this price–performance linkage is critical. In various regions globally, the practice of prescribing generic medications is widely embraced.^14^ However, this approach has not gained traction in some countries, including Tanzania, primarily due to issues like multiple circulating brands in the market and scepticism regarding product quality.^15^ In countries with limited resources, obtaining an affordable antimicrobial agent for treatment of the same illness could substantially lower treatment costs.^8,16^ This has far-reaching implications for local practices, formulary decisions, generic substitution policies, and global efforts to enhance rational, equitable antibiotic use.^16^

This study aimed to rule out the possibility of circulating sub-standard ceftriaxone brands in Tanzania, which bear the risk of exerting selective pressure on the microbial population.

We intended to bridge the knowledge gap by empirically investigating correlations between cost and performance across ceftriaxone brands in treating a ceftriaxone-sensitive uropathogenic *E. coli*. By comparing the effectiveness of these antibiotics against isolated bacterial strains in a controlled setting, we endeavour to produce *in vitro* data for potential *in vivo* studies that can provide a scientific basis for prescribing practices.

## Materials and methods

### Study design

This was an experimental study carried out in July 2023 at the microbiology laboratory of our hospital, a tertiary public institution situated in Tanzania’s Southern Highlands. We assessed various locally available ceftriaxone brands obtainable from private pharmacies in Mbeya Central Business District (CBD), Tanzania.

Five ceftriaxone brands were purchased with the following brand names: Cefgen (Reyoung Pharmaceutical Company Limited, China), Epicephin (Egyptian International Pharmaceutical Industries Company, Egypt), Trixone [Reyoung Pharmaceutical Company Limited, China, for Abacus Pharma(A) Limited, East Africa], Mesporin (Labesfal-Laboratorios Almiro, S.A., Portugal, for Acino AG, Germany), Alcef (Scott-Edil Advance Research Laboratories and Education Ltd, India).

Each ceftriaxone brand was allocated a unique laboratory identifier to ensure anonymity throughout the laboratory procedure and statistical analysis. Two active batches from each brand were incorporated into the study. The cost for 1 g of ceftriaxone from these brands spanned from TZS 700 to TZS 22 000, as detailed in Table 1.

**Table 1.** Ceftriaxone brands included in the study and their prices

Lab identifier	Batch no.	Brand name	Mfg. date	Exp. date	Price in TZS^a^
1	U0122U0139	Mesporin 1 g	07/2020	07/2023	22 000/=
1B	X0075	Mesporin 1 g	06/2021	06/2024	
3B	13422005	Alcef 1 g	01/2022	12/2024	1500/=
3C	13422042	Alcef 1 g	03/2022	02/2025	
4B	2102102	Epicephin 1 g	02/2021	02/2024	10 000/=
4C	2203611	Epicephin 1 g	03/2022	03/2025	
5B	223051009	Cefgen 1 g	01/2022	12/2024	700/=
5C	213051256	Cefgen 1 g	11/2021	10/2024	
7B	220323	Triaxone 1 g	03/2022	02/2025	1500/=
7C	220321	Triaxone 1 g	03/2022	02/2025	

^a^TZS standards for Tanzanian shillings (1 USD = 2500 TZS).

### Laboratory procedures

Each batch was serially diluted to a concentration of 62.5 mg/L. A freshly overnight-grown ceftriaxone-sensitive *E. coli* inoculum was prepared in a distilled water tube to make a 0.5 McFarland concentration. For each brand, 100 μL of Mueller–Hinton broth was added in rows of 12 microwells, followed by 100μL of 62.5 mg/L ceftriaxone into the first well of each row and mixed to make a concentration of 31.25 mg/L. A serial dilution was then performed by transferring and mixing 100 μL from each well into the next until the 10th well, resulting in concentrations of 31.25, 15.66, 7.81, 3.91, 1.95, 0.98, 0.49, 0.24, 0.12, and 0.06 mg/L for each brand. Then, 100 μL of the 0.5 McFarland *E. coli* inoculum was added to each well except the 11th column in which 100 μL of distilled water was added to serve as a negative control and the 12th column served as a positive control. The microwell plates were covered and incubated overnight at 37°C. Finally, the Sunrise™ Plate Reader (Tecan, Switzerland) was used to determine the optical densities of each microwell at 450 nm wavelength to assess bacterial growth inhibition.

The same procedure was repeated for the second run to make a total of four tests per each brand.

### Statistical analyses

We started by plotting the mean rate of clearing bacteria in the cultured samples for each of the brands at different concentration levels. We then performed an analysis of variance (ANOVA) to test if there was a significant difference in performance among different ceftriaxone brands. All analyses were performed using Excel and SPSS version 29. A *P* < 5% was considered statistically significant.

### Ethics approval

This study was conducted in accordance with the Declaration of Helsinki and had been reviewed and approved by the Mbeya Medical Research and Ethics Committee (MMReC) (ref. no. SZEC-2439/RA/V.1/92a).

## Results

### Performance of different ceftriaxone brands

**Figure 1.**
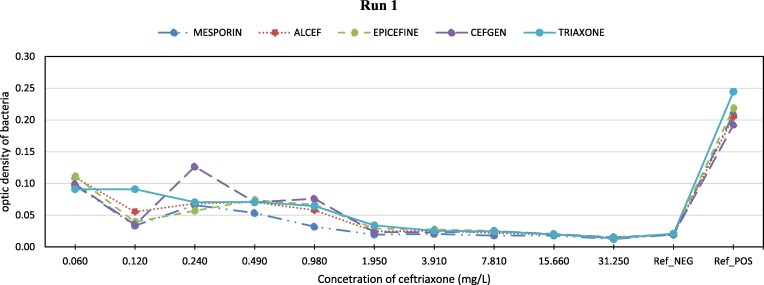
Optic density of bacterial growth vs. ceftriaxone concentration (mg/L).

**Figure 2.**
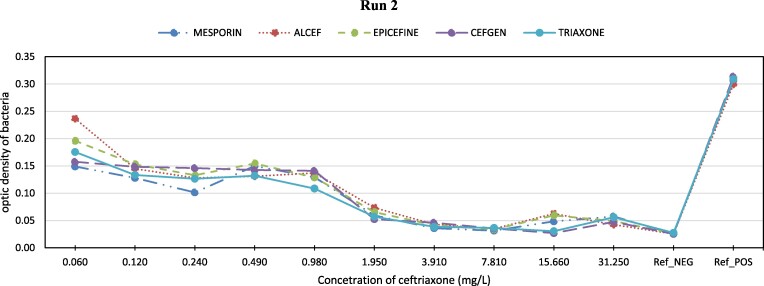
Optic density of bacterial growth vs. ceftriaxone concentration (mg/L).

**Figure 3.**
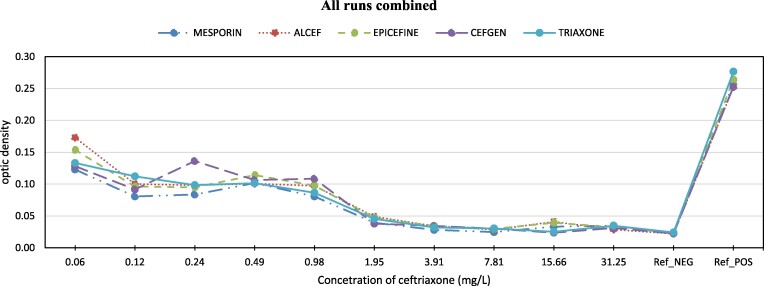
Optic density of bacterial growth vs. ceftriaxone concentration (mg/L) - combined data from all runs.

The performance of the different ceftriaxone brands at each concentration level is displayed in Figures 1–3. Across the concentration range, all brands demonstrated a similar pattern of bacterial growth inhibition. At higher concentrations above 1 mg/L, the performance was high with near complete clearance of bacteria for all brands. As the concentration decreased, more bacterial growth was observed. However, the growth rate did not differ between brands at the same concentration. The optimal performance, defined as clearance comparable to the negative control, was seen between concentrations of 1.95–31.25 mg/L for all brands. At 1.95 mg/L concentration, bacterial inhibition reached the same level as the negative control for most brands. Between 3.91 and 7.81 mg/L, complete inhibition matched the negative control for all brands tested.

### Comparison of the performance of different ceftriaxone brands

Table 2 illustrates that ANOVA analysis found no significant differences among brands across tested concentrations (*P* values near 1). Additionally, a negligible mean square error (MSE) (<0.001) at all levels suggests consistent performance across brands, corroborated by similar inhibition patterns in Figure 1.

**Table 2.** ANOVA for the performance of ceftriaxone brands

Concentration (mg/L)	ANOVA component	SSE	df	MSE	*F*	*P* value
31.25	Between brands	<0.001	4	<0.001	0.035	0.997
	Within brands	0.011	15	0.001		
	Total	0.012	19			
15.66	Between brands	0.001	4	<0.001	0.643	0.640
	Within brands	0.006	15	<0.001		
	Total	0.007	19			
7.81	Between brands	<0.001	4	<0.001	0.420	0.791
	Within brands	0.001	15	<0.001		
	Total	0.001	19	<0.001		
3.91	Between brands	<0.001	4	<0.001	0.274	0.890
	Within brands	0.002	15	<0.001		
	Total	0.002	19	<0.001		
1.95	Between brands	<0.001	4	<0.001	0.180	0.945
	Within brands	0.008	15	0.001		
	Total	0.008	19			
0.98	Between brands	0.002	4	<0.001	0.256	0.902
	Within brands	0.027	15	0.002		
	Total	0.029	19			
0.49	Between brands	0.001	4	<0.001	0.061	0.992
	Within brands	0.032	15	0.002		
	Total	0.032	19			
0.24	Between brands	0.006	4	0.002	1.251	0.332
	Within brands	0.019	15	0.001		
	Total	0.025	19			
0.12	Between brands	0.002	4	0.001	0.175	0.948
	Within brands	0.046	15	0.003		
	Total	0.048	19			
0.06	Between brands	0.007	4	0.002	0.626	0.651
	Within brands	0.041	15	0.003		
	Total	0.048	19			
Ref_NEG	Between brands	<0.001	4	<0.001	0.191	0.939
	Within brands	<0.001	15	<0.001		
	Total	<0.001	19	<0.001		
Ref_POS	Between brands	0.002	4	<0.001	0.133	0.968
	Within brands	0.046	15	0.003		
	Total	0.048	19			

SSE, sum of square error; MSE, mean square error; df, degree of freedom; *F*, Fisher’s test.

## Discussion

The primary objective of this study was to compare the bacterial inhibition performance of different brands of ceftriaxone at varying concentrations. Notably, all brands demonstrated comparable bacterial inhibition capabilities. Similar results were observed in research conducted in Afghanistan, which compared the *in vitro* efficacy of 40 brands against *Staphylococcus aureus*, ATCC 292.^17^ However, our findings partly differ from those in a Nepal study, which compared only 3 brands but 13 clinical isolates and found that 2 brands were consistent, but 1 had at least 2-fold higher MIC.^18^ The difference with the Nepal study could be accounted for by the difference in the methodology, particularly the number of isolates tested; also, the types tested and the brands were not declared.

The results of our study evidence uniformity in performance, showing no statistically significant variations among brands in their rate of bacterial inhibition at any concentration. This finding has important clinical implications, as it assures clinicians that they can confidently prescribe different brands of ceftriaxone while expecting consistent outcomes. Therapeutic decisions need not be constrained by brand availability or cost, as performance appears preserved across brands. Additionally, the high potency of ceftriaxone at higher concentrations was consistently observed.

The ANOVA results provide further statistical support for the observed uniform performance of ceftriaxone brands. The *P* values approaching 1 and negligible MSE between groups indicate strong evidence against any significant performance differences between brands. Though this study was limited in scope, particularly of pathogenic bacterial strains, the consistency of results across the brands and concentrations tested gives credibility to the findings. Apart from clinical relevance, these results have important economic implications. Despite more than 30-fold price differences, the high performance demonstrates the absence of a clear price–performance correlation for ceftriaxone brands. This suggests the substantially higher cost of some brands is not justified by superior quality or effectiveness.

The Tanzania Medicine and Medical Devices Authority (TMDA) in corporation with the Tanzania Police Force, Weights and Measures Agency, Tanzania Fair Competition Commission, Tanzania Bureau of Standards, and Tanzania Revenue Authority Customs leads in the fight against counterfeit/sub-standard drugs in Tanzania.^3^

Our findings on the performance of different ceftriaxone brands in Mbeya Market may, therefore, have a far-reaching implication countrywide and is a credit to TMDA. However, this kind of study should be conducted regularly for different clinical bacteria to inform and assure clinicians, patients, and healthcare facilities.

Despite impressive findings, some limitations of this study include the small sample size, lack of diverse bacterial specimens, and inability to evaluate clinical outcomes. Testing was restricted to a single uropathogenic *E. coli* isolate, and only optical density was used to quantify bacterial growth inhibition. While optical density serves as a proxy for antibacterial activity, additional quantitative microbiological assays could have provided more robust results. Furthermore, bioequivalence between brands was not examined, so differences in pharmacokinetic parameters could influence clinical effectiveness despite similar *in vitro* performance.

This study found no statistically significant differences in the *in vitro* antibacterial performance of the five ceftriaxone brands tested against an *E. coli* isolate. Across the concentration range, all brands demonstrated comparable bacterial inhibition profiles. We found no significant differences between brands at any concentration. These findings indicate there is no correlation between the price and *in vitro* performance of ceftriaxone brands available in Mbeya City. The high cost of a brand may not imply superior quality or effectiveness. *In vivo* studies can answer whether clinicians can prescribe different ceftriaxone brands interchangeably, allowing choice based on availability and affordability. Luckily in Tanzania, all choices are equally available in the market. Further research would help substantiate the generalizability of these findings and their implications for clinical practice and health policy.

## Data Availability

All data generated or analysed during this study are included in this published article.
